# Factors correlated with drug use for constipation: perspectives from the 2016 open Japanese National Database

**DOI:** 10.1186/s12876-020-01425-6

**Published:** 2020-08-24

**Authors:** Hiroshi Mihara, Aiko Murayama, Sohachi Nanjo, Takayuki Ando, Kazuto Tajiri, Haruka Fujinami, Masaaki Yamada, Ichiro Yasuda

**Affiliations:** 1 Department of Gastroenterology, Graduate School of Medicine and Pharmaceutical Sciences, University of Toyama, Sugitani, Toyama, 2630 Japan; 2 Center for Medical Education and Career Development, Graduate School of Medicine and Pharmaceutical Sciences, University of Toyama, Toyama, Japan; 3 Department of Epidemiology and Health Policy, Graduate School of Medicine and Pharmaceutical Sciences, University of Toyama, Toyama, Japan

**Keywords:** Low outside temperature, ecological analysis, chronic constipation, laxatives

## Abstract

**Background:**

The prevalence of chronic constipation is increased in females and with age or environmental (low temperature), racial, socioeconomic, and habitual risk factors. The impact of low outside temperature on constipation drug use remains unclear. Here, we investigated risk factors for constipation drug use by evaluating data from the Japanese National Database.

**Methods:**

This ecological study used the 2016 open Japanese National Database of health insurance claims (prescriptions) to acquire the number of health insurance prescription claims in all 47 prefectures for drugs to relieve constipation, antihypertensives, vasodilators, as well as medical check-ups and questionnaire responses. Internet survey on room temperatures in 2010 were also used. Pearson correlation coefficients (r) between the number of population-based prescriptions for each item were calculated and multiple linear regression analysis (MLR) was performed.

**Results:**

Prescriptions for magnesium laxatives significantly correlated with aging (*r* = 0.58), vasodilators (*r* = 0.53), being female (*r* = 0.43), antihypertensives (*r* = 0.39), and inversely with eating ≤2 h before bedtime (*r* = − 0.37), total crime rate (*r* = − 0.33), insomnia (*r* = − 0.33), and population density (*r* = − 0.31). Stimulant laxatives (sennoside and picosulfate) were significantly correlated with antihypertensives (*r* = 0.79), aging (*r* = 0.69), vasodilators (*r* = 0.67), and being female (*r* = 0.56), and were inversely associated with average outside temperature (*r* = − 0.62), total crime rate (*r* = − 0.52), average income (*r* = − 0.51), and 30-min of vigorous exercise (*r* = − 0.44). Fecal interventions were significantly correlated with aging (*r* = 0.55) and female (*r* = 0.59), and inversely correlated with population density (*r* = − 0.41) and total crime rate (*r* = − 0.38). MLR analysis identified aging as the only significant risk factor for magnesium laxative use (partial slope [β] = 1241.0). Female sex and antihypertensives were independent risk factors for stimulant laxative prescriptions (β = 44,547.0 and 0.2) and average outside temperature and 30-min of vigorous exercise were independent preventive factors (β = − 616.8 and − 219.1).

**Conclusion:**

We identified associations of magnesium laxatives with aging, stimulant laxatives with female sex, antihypertensives, low outside temperature and less 30 min of vigorous exercise.

## Background

Although the prevalence of chronic constipation varies in different studies (2–27%) [1], the 2017 National Survey of Basic Life Surveys in Japan reported that the rate of constipation-related complaints was 2.5% in males and 4.6% in females [2]. Studies in Japan and the United States have shown that the prevalence of constipation increases with age in both sexes and is higher in females [1, 3]. A higher prevalence of constipation has also been reported in non-whites than in whites, and in those with fewer years of education or a lower socioeconomic status [1]. With regard to risk factors, no relationship between body mass index (BMI) and constipation has been observed, although eating significantly less food at breakfast has been reported in constipated children [4, 5]. While moderate exercise does not alter intestinal function, vigorous exercise such as jogging is considered to enhance transit through the digestive tract [6]. There is evidence that vigorous exercise can relieve constipation in the elderly [7–9]. Sleeplessness, or staying awake for long periods of time, is also reported among people with constipation [10, 11]. Johanson (1998) was the first to report that a low temperature was one of the global environmental risk factors for constipation [12]. In contrast, there are no reports of risk factors for each constipation drug used by the population, or based on climate, and socioeconomic factors. The open Japanese National Database (NDB) was publicly released to researchers in 2011, and the number of studies using the NDB has grown rapidly [13]. The aim of this study was to investigate risk factors for constipation drug use with an emphasis on residential and environmental factors using the open Japanese National Database (NDB).

## Methods

### Study type

Ecological study.

### Data source (for this secondary data source)

Data were obtained from the 2016 open Japanese NDB which was published by the Ministry of Health, Labor and Welfare [14]. Data pertaining to the population (age [> 65 years], sex, population density, and total crime rate), climate (average outside temperature and humidity, precipitation in capital cities, and mean elevation above sea level), and socioeconomic factors (average monthly and total family income) for all 47 prefectures in Japan in 2016 were obtained from the online database of the Ministry of Internal Affairs and Communications and Geospatial Information Authority of Japan [15, 16].

### Data extraction

The extracted data from NDB included the following: (1) the number of population-based health insurance claims (prescriptions) for prescription medicines (e.g., laxatives including magnesium-based agents, stimulant laxatives, lactulose, herbal medicines, antihypertensives, and vasodilators), (2) fecal interventions (e.g., enemas, disimpaction treatments), (3) prescriptions for suppositories and enemas, (4) specific medical check-ups (e.g., for BMI, hemoglobin A1c, and abdominal circumference measurements), (5) responses to questionnaires (e.g., related to insomnia, antihypertensives, smoking, drinking, eating, and exercise habits) and (6) psychiatric specialty therapy.

### Inclusion and exclusion criteria

Magnesium sulfate, which was originally classified for laxative and enema use, was categorized as a magnesium laxative for the purposes of the analysis in this study. Antihypertensives included angiotensin II receptor blockers (e.g., telmisartan), 1 blockers (e.g., doxazosin), blockers (e.g., carvedilol), angiotensin-converting enzyme inhibitors (e.g., enalapril), and some calcium (Ca) blockers (e.g., amlodipine) and vasodilators, including nitrate (e.g., isosorbide mononitrate). To minimize ecological fallacy, the extent and severity to which constipation affected the population were determined using the number of health insurance claims (prescriptions) for the treatment of constipation per 1000 people. The above-reported risk factors of constipation were included as variables (sex, aging, environment, socioeconomic status, habits, and some constipating drugs [e.g., antihypertensives and vasodilators]). Low outside temperature was found to be a significant risk factor for constipation drug use. However, because room temperature is not always associated with outside temperature, depending on location, we searched inside room temperature in 2016 to determine which temperature (outside or inside) was the dominant factor affecting constipation drug use; inside room temperature in 2016 was not available. Because the correlation coefficient of outside temperature between 2010 and 2016 was adequately high (R^2^ = 0.99), we decided to use inside room temperature in 2010 [17], combined with the 2016 dataset. Lactulose is approved for not only constipation but also hepatic hyperammonemia and defecation for gynecologic surgery in Japan. Moreover, because we could not differentiate lactulose for constipation from other indications, we decided to exclude all lactulose from this study.

### Sampling technique employed

Cluster sampling (Japanese population and/or specific medical check-ups; examinees were between 40 and 74 years old in 2016).

#### Statistical analysis and other

SPSS Statistics 24.0 (IBM Corp., Armonk, NY) was used to calculate correlation coefficients between the number of population-based insurance claims (prescriptions) per 1000 people for each drug for constipation. Statically significant variables (*p* < 0.05) were extracted for multiple linear regression (MLR) analysis. MLR was subsequently performed using a stepwise method to exclude the confounding variables and identify the independent predictors of constipation drug use.

## Results

Table 1 shows the means and SDs of population, climate, and socioecological variables in 47 Japanease prefectures. The mean rate of Japanese people who were > 65 years (aging) was 29.6%. The Japanese population had slightly more females than males. The average number of insurance claims (prescriptions) summarized by constipation and cardiovascular drug use by people is shown in Table 2. In this study lactulose, herbal medicines, and novel drugs (e.g., lubiprostone) were excluded from the analysis. Pearson correlation coefficients (r) are shown in Table 3. Prescriptions for magnesium laxatives significantly correlated with aging (*r* = 0.58), vasodilators (*r* = 0.53), female sex (*r* = 0.43), and antihypertensives (*r* = 0.39) and were inversely correlated with eating ≤2 h before bedtime (*r* = − 0.37), total crime rate (*r* = − 0.33), insomnia (*r* = − 0.33), and population density (*r* = − 0.31), etc.

**Table 1:** Variables assessed in the population (*N* = 47 Japanese prefectures)

**Variables**	**Mean**	**± SD**
Age, > 65 years (%)	29.6	2.9
Female: Male	1:1	0.0
Population density (km^2^)	655.3	1194.4
Total crimes/10 months per 1000 people	5.4	1.8
Average outside temperature (°C)	16.2	2.4
Average room temperature (°C)	18.8	0.9
Average humidity (%)	70.6	4.2
Precipitation in capital city (mm/year)	1609.2	418.0
Mean elevation above sea level (m)	361.1	216.8
Average income (thousand yen)		
Males	298.3	28.9
Females	211.5	19.0
Total household	5189.1	520.6
Total worker household	6031.0	549.4
Obese, BMI > 25 kg/m^2^ (%)	26.7	2.6
Weight loss, BMI < 20 kg/m^2^ (%)	17.8	1.7
Insomnia (%)	34.3	4.1
Hyperglycemia (%)	7.0	0.6
Antihypertensive drug use (%)	21.5	2.3
Waist circumference > 90 cm (%)	21.1	1.7
Smoking (%)	22.7	1.9
Daily alcohol intake (%)	27.8	2.2
No breakfast (%)	14.6	2.4
Eating after dinner (%)	15.9	2.3
Eating ≤2 h before bedtime (%)	25.8	2.7
Eating rapidly (%)	31.1	1.8
Walking fast (%)	45.0	4.1
Walking > 1 h (%)	39.9	5.5
30-min of vigorous exercise (%)	27.2	3.3
Psychiatric specialty therapy per 1000 people	891.7	219.7

*BMI* body mass index

**Table 2:** Health insurance claims (prescriptions) among residents in all Japanese prefectures (*N* = 47)

	**Average claims (prescriptions) per 1000 people, n**	**± SD**	**Minimum**	**Maximum**
Constipation drugs				
Magnesium	33,431	6095	19,537	43,859
Stimulant laxatives	16,603	4251	8181	31,672
Lactulose	3555	820	820	5420
Herbal medicines	10,333	1826	1826	16,475
Fecal interventions	37	11	18	63
Suppositories and enemas	131	60	53	292
Cardiovascular drugs				
Antihypertensives	49,196	6805	34,440	71,422
Vasodilators	47,037	6990	34,214	61,601

**Table 3:** Relationships between variables and drugs to relieve constipation (*N* = 47 prefectures in Japan)

	**Pearson correlation coefficients by type of drug used to relieve constipation**			
	**Magnesium**	**Stimulant laxative**	**Fecal intervention**	**Suppositories and enemas**
Aging (> 65 years)	0.583^**^	0.693^**^	0.552^**^	0.303^*^
Vasodilator claims/prescriptions (per 1000 people)	0.526^**^	0.667^**^	0.308^*^	0.2
Female gender	0.434^**^	0.562^**^	0.592^**^	0.453^**^
Antihypertensive claims/prescriptions (per 1000 people)	0.389^**^	0.794^**^	0.338^*^	0.1
Eating ≤2 h before bedtime	−0.366^*^	− 0.288^*^	− 0.372^**^	− 0.1
Total crimes/10 month (per 1000 people)	− 0.334^*^	− 0.515^**^	− 0.386^**^	− 0.2
Insomnia	− 0.327^*^	− 0.2	− 0.326^*^	− 0.1
Population density (per km^2^)	−0.308^*^	− 0.348^*^	− 0.405^**^	− 0.2
Average humidity	0.293^*^	0.353^*^	0.310^*^	0.2
Psychiatric specialty therapy (per 1000 people)	0.2	0.2	0.333^*^	0.3
Antihypertensives	0.2	0.424^**^	0.1	0.2
Daily alcohol intake	0.1	0.511^**^	0.3	−0.1
Eating after dinner	0.1	0.2	0.291^*^	0.344^*^
Mean elevation above sea level	0.1	0.0	0.2	0.0
Eating rapidly	0.1	−0.2	0.1	0.317^*^
Weight loss (BMI < 20 kg/m^2^)	0.0	−0.2	0.2	−0.2
30-min of vigorous exercise	0.0	−0.442^**^	− 0.288^*^	0.2
Average outside temperature	0.0	−0.622^**^	−0.2	0.1
Hyperglycemia	0.0	0.2	0.1	0.2
Precipitation in capital city	−0.1	−0.1	0.0	−0.1
Waist circumference > 90 cm	−0.1	− 0.1	−0.2	0.1
Obese (BMI > 25 kg/m^2^)	−0.1	0.1	−0.2	0.1
Total worker household income	−0.2	−0.2	− 0.299^*^	−0.355^*^
Walking > 1 h	− 0.2	− 0.1	− 0.1	− 0.1
Average room temperature	−0.2	0.364^*^	0.1	−0.1
Total household income	−0.2	− 0.2	− 0.3	−0.353^*^
Smoking	−0.2	0.489^**^	0.0	− 0.3
Female average income	−0.2	− 0.522^**^	− 0.343^*^	−0.2
No breakfast	−0.2	− 0.334^*^	− 0.2	0.0
Walking fast	−0.2	−0.2	− 0.2	0.1
Male average income	−0.3	− 0.513^**^	−0.380^**^	− 0.3

*BMI* body mass index

*p < 0.05, **p < 0.01

Stimulant laxative prescriptions were significantly correlated with antihypertensive drug uses (*r* = 0.79), aging (*r* = 0.69), vasodilator prescriptions (*r* = 0.67), female sex (*r* = 0.56), daily alcohol intake rate (*r* = 0.51), smoking rate (*r* = 0.49), antihypertensive prescriptions (*r* = 0.42), average room temperature (*r* = 0.36), and average outside humidity (*r* = 0.35) etc., and inversely correlated with average outside temperature (*r* = − 0.62), total crime (*r* = − 0.52), average income (*r* = − 0.51), 30-min of vigorous exercise (*r* = − 0.44), population density (*r* = − 0.35), no breakfast (*r* = − 0.33), and eating ≤2 h before bedtime (*r* = − 0.29), etc.

For fecal interventions, significant positive correlations were observed with aging (*r* = 0.55), female sex (*r* = 0.59), average outside humidity (*r* = 0.31), antihypertensives (*r* = 0.34), and psychiatric specialty therapy per 1000 people (*r* = 0.33). Negative correlations were noted for population density (*r* = − 0.41), total crime rate (*r* = − 0.38), average incomes (*r* = − 0.38 and − 0.34), eating ≤2 h before bedtime (*r* = − 0.37), insomnia (*r* = − 0.33), and 30-min of vigorous exercise (*r* = − 0.29).

Prescriptions for suppositories and enema correlated significantly with female sex (*r* = 0.45), total household income (*r* = 0.35), and eating after dinner (*r* = 0.34), etc.

Overall, risk factors for constipation-related treatments included population factors (aging, female sex, daily alcohol intake, smoking, antihypertensives, and eating after dinner) and climate factors (average outside humidity and average room temperature). In contrast, preventive factors for constipation-related treatments included factors related to the population (eating ≤2 h before bedtime, 30-min of vigorous exercise, and no breakfast), climate (outside temperature), and socioecological aspects (total crime rate, population density, and average incomes). As the difference of the values of these factors must be dependent on the place of residence and dietary habits that must differ by location, to exclude these confounding factors, MLR was performed using each significant variable that appeared explanatory.

**Fig. 1:**
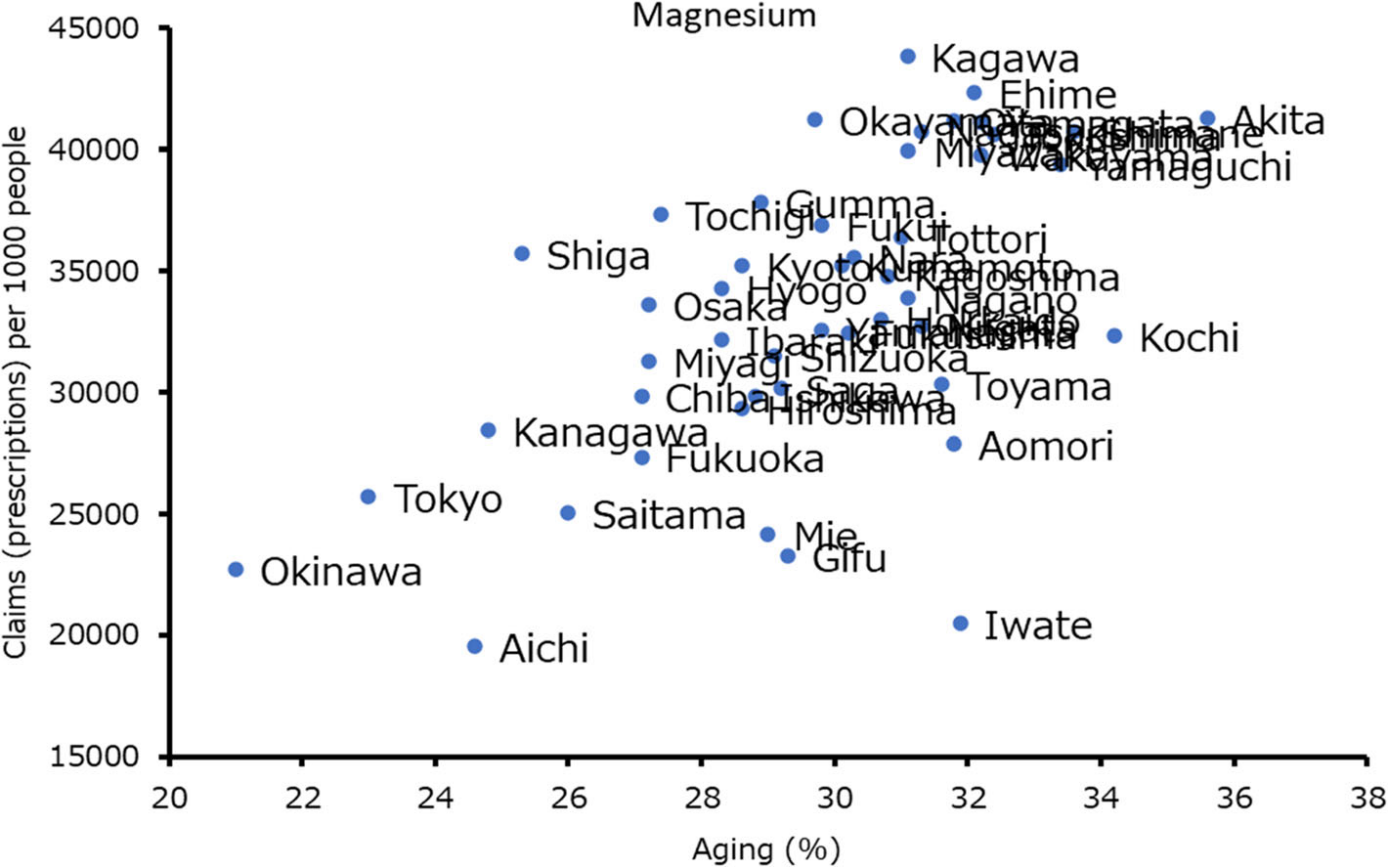
Aged persons (%) and magnesium prescription claims (prescriptions) per 1000 people

**Fig. 2:**
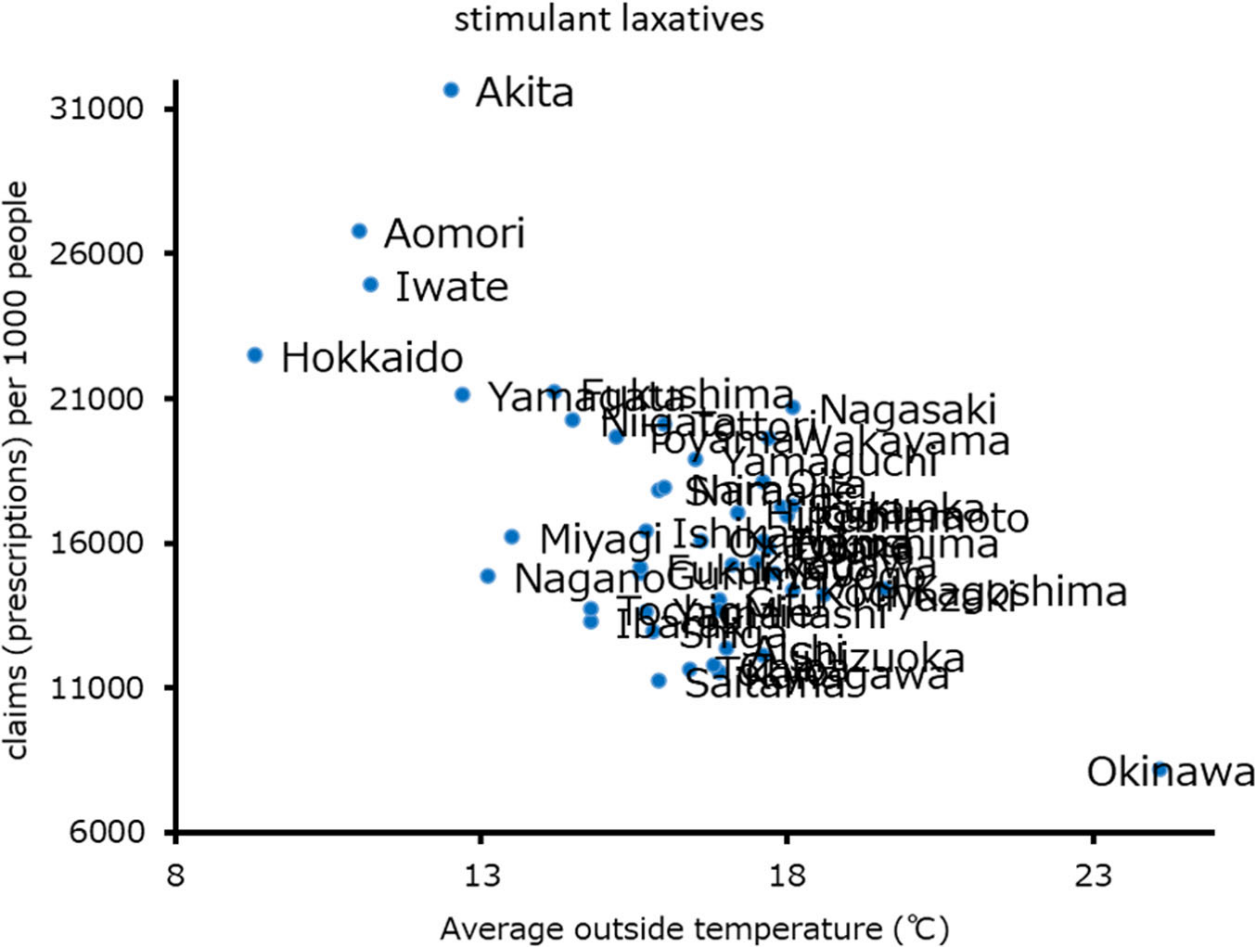
Average outside temperature and stimulant laxative prescriptions claims (prescriptions) per 1000 people

Aging was observed to be the only significant risk factor for the use of magnesium laxatives (F-value 23.2, R^2^ 0.34, partial slope [β] = 1241.0; Table 4). Plotted data between aging and magnesium claims (prescriptions) per 1000 people is shown in Fig. 1. Female sex and antihypertensives were independent risk factors for stimulant laxative prescriptions (β =44,547.0 and 0.2), average outside temperature and 30-min of vigorous exercise were independent preventive factors (β = − 616.8 and − 219.1) but not room temperature (F-value 4.9, R^2^ 0.81, Table 5). Plotted data between average outside temperature and stimulant laxative claims per 1000 people are shown in Fig. 2. Female sex was an independent risk factor for fecal interventions (β =146.8). Insomnia and 30-min of vigorous exercise were observed to be independent preventive factors for fecal interventions (β = − 0.7 and − 1.2; F-value 4.5, R^2^ 0.50, Table 6).

**Table 4:** Multiple linear regression analysis for magnesium laxatives

	**Partial slope [β]**	***p*** **value**	**95.0% Confidence interval**	
			**Lower limit**	**Upper limit**
Aging	1241.0	0.00**	722.0	1759.9

**p < 0.01

**Table 5:** Multiple linear regression analysis for stimulant laxatives

	**Partial slope [β]**	***p*** **value**	**95.0% Confidence interval**	
			**Lower limit**	**Upper limit**
Female gender	44,547.0	0.00**	27,991.2	61,102.8
Antihypertensive claims (prescriptions) per 1000 people	0.2	0.00**	0.09	0.34
Average outside temperature	−616.8	0.00**	− 958.4	− 275.1
30-min of vigorous exercise	−219.1	0.03*	− 419.6	− 18.6

*p < 0.05, **p < 0.01

**Table 6:** Multiple linear regression analysis for fecal interventions

	**Partial slope [β]**	***p*** **value**	**95.0% confidence interval**	
			**Lower limit**	**Upper limit**
Female gender	146.8	0.00**	85.5	208.1
Insomnia	−0.7	0.04*	−1.3	−0.0
30-min of vigorous exercise	−1.2	0.00**	−1.9	−0.4

* *p* < 0.05, ** *p* < 0.01

Female sex was an independent risk factor for prescriptions for suppositories and enemas (β = 656.5; F-value 11.6, R^2^ 0.21, Table 7).

**Table 7:** Multiple linear regression analysis for suppositories and enemas

	**Partial slope [β]**	***p*** **value**	**95.0% confidence interval**	
			**Lower limit**	**Upper limit**
Female gender	656.5	0.00**	268.4	1044.5

**p < 0.01

## Discussion

Here, we report the results of an ecological analysis of health insurance claims (prescriptions) for the alleviation of constipation using the Japanese NDB dataset. Previous studies have reported the association of aging with constipation [1, 3]. Foremost, our results showed that aging was associated with the number of magnesium laxative prescriptions but not the number of other drugs (Tables 4, 5, 6 and 7). To the best of our knowledge, no reports have described the responses of age-related constipation to magnesium laxatives, particularly with regard to the effects of risk factors other than aging. Female sex was an independent risk factor for the use of stimulant laxatives, fecal interventions, and prescriptions for suppositories and enemas, consistent with previous reports [1, 3]. Johanson was first to report that low temperatures were one of the global environmental risk factors for constipation [12]. In this study, we showed that outside temperature and vigorous exercise were independent preventive factors for stimulant laxative prescriptions (Table 5). However, the mechanisms through which outside temperature and vigorous exercise affect stimulant laxative use are not clear. In previous reports, internal body temperature has been shown to be synchronized with outside temperature [18, 19], increased with metabolic rate in dogs and humans [20, 21] and with vigorous exercise [22], and decreased with aging [23]. Additionally, intestinal contraction or transit may affect in low internal temperature in humans, as demonstrated in an ex-vivo study [24] and may improve with vigorous exercise or external body warming using lumbar application of a 40 °C hot compress in women with constipation [22, 25]. These reports have suggested that aging may be a necessary risk factor and that low outside temperature could be an added factor augmenting the need for stimulant laxatives. Antihypertensives were also an independent risk factor for stimulant laxative prescriptions. Antihypertensive use, rather than vasodilator use, may be a risk factor for constipation drug use. In this study, there were no associations between use of drugs to relieve constipation and underlying diseases (obesity, weight loss, insomnia, hyperglycemia, and psychiatric specialty therapy) and socioeconomic status (income and educational environment).

There were some limitations in this study. The first was that the number of health insurance claims (prescriptions) related to prescriptions for the treatment of constipation were used as a surrogate measure of the extent and severity to which constipation affected the population. And in this study lactulose, herbal medicines, and several novel drugs available from 2012 were excluded. This type of ecological study has inherent problems related to extrapolation of relationships among groups to individuals. We considered using other statistical analysis including multilevel modelling (MLM) techniques, which would overcome the ecological fallacy and help to examine the contextual effects. However, because they required information at the individual level, we thought that MLM and other analysis could not be applied in this study. The second limitation is that the room temperatures (not an independent risk factor) used in this analysis were derived from 2010 data sourced via an internet search [17]. In this study, we evaluated the correlation coefficient of outside temperature between 2010 and 2016 (R^2^ = 0.99, Supplementary Fig. 1). Our result supported the validity of using inside room temperature in 2010 in our analysis of the 2016 dataset. The third limitation is that this study was based on 2016 data; however, it is the latest available data set of its kind. Finally, some potential confounding factors (e.g., other constipation-related diseases or drug use) were not included in our analysis.

## Conclusion

In this study, we performed an ecological analysis of drugs used to alleviate constipation in the Japanese population. Our findings showed that magnesium-based mediation alone was associated with aging and that stimulant laxative use was associated with female sex, lower outside temperature, antihypertensives, and vigorous exercise. Further studies are required to evaluated the causative relationships among these factors.

## Supplementary information

**Additional file 1.**

**Additional file 2.**

**Additional file 3: Supplementary Fig. 1.** Correlation coefficient of outside temperature between 2010 and 2016.

## Abbreviations

NDB: National Database
MLR: Multiple linear regression analysis
