# Supplementary material for: Factors correlated with drug use for constipation: perspectives from the 2016 open Japanese National Database
Source: BMC Gastroenterol. 2020 Aug 24;20:284. doi: 10.1186/s12876-020-01425-6 (PMC7444268; doi:10.1186/s12876-020-01425-6)
Supplement: Supplementary file 3 — Additional file 3: Supplementary Fig. 1. Correlation coefficient of outside temperature between 2010 and 2016. [file 12876_2020_1425_MOESM3_ESM.docx]

**Supplementary Figure 1.** Correlation coefficient of outside temperature between 2010 and 2016

**Supplementary Fig. 1**

**
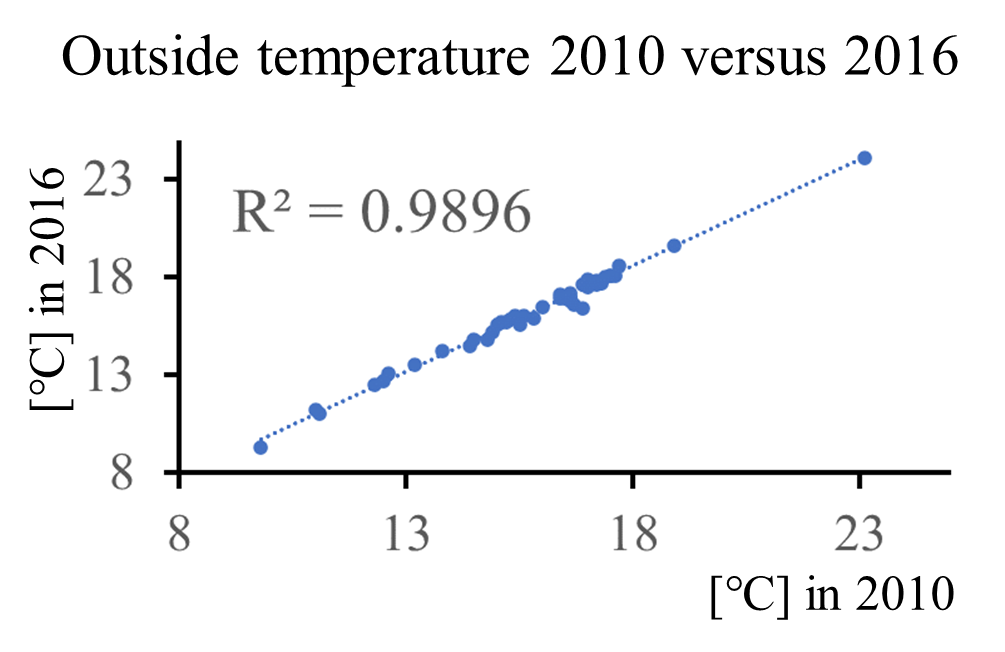
**
